# The Generational Impact on Meaning Making and Well-being of Adult Children Caregivers in Dementia Caregiving

**DOI:** 10.1093/geroni/igaa057.3341

**Published:** 2020-12-16

**Authors:** Vivian Lou, Daniel W L Lai, Daniel Fu-Keung Wong, Doris Yu, Shuangzhou Chen, Reynold Leung

**Affiliations:** 1 The University of Hong Kong, Hong Kong, China; 2 Hong Kong Baptist University, Hong Kong, China

## Abstract

Children caregivers contributed significantly to care and support dementia parents globally. In the caregiving journey, making sense of providing care plays significant role in their caregiving journey. In an ageing society such as Hong Kong, different generations of children caregivers take up dementia caregiver roles. We hypothesized that from studying baby boomers (BB, born in 1946-1964) and generation X (GX, born in 1965-1980), generations have impacts on their meaning making and well-being outcomes. 601 Caregivers completed a paper or online battery of questionnaires on burden (ZBI-4), mental well-being (PHQ-9), caregiving factors (ADL, IADL, caregiving hours, Positive Aspect of Caregiving; PAC) and the meaning making factors (Finding Meaning Through Caregiving; FMTC). Results showed that significant difference between caregivers from two generations. GX have significantly lower meaning made, measured by PAC affirming self and enriching life, as well as FMTC provisional meaning. While they spent less caregiving hours for the more independent care recipients, they suffered from higher burden, higher FMTC loss/powerless and worse psychological well-being (PHQ). The findings demonstrated generation X caregiver suffered from lower level of the meaning made and worse psychological wellbeing outcomes than BB caregivers. Future caregiver studies should take generational effect into account and services shall be provided in a generation-responsive approach.

